# Spectral-domain optical coherence tomography assessment of retinal and choroidal changes in patients with coronavirus disease 2019: a case-control study

**DOI:** 10.1186/s12348-022-00297-z

**Published:** 2022-06-18

**Authors:** Mojtaba Abrishami, Ramin Daneshvar, Zahra Emamverdian, Neda Saeedian, Fariba Tohidinezhad, Saeid Eslami, Mohammad-Reza Ansari-Astaneh

**Affiliations:** 1 Eye Research Center, Mashhad University of Medical Sciences, Mashhad, Iran; 2 Department of Internal Medicine, Faculty of Medicine, Mashhad University of Medical Sciences, Mashhad, Iran; 3 Department of Medical Informatics, Faculty of Medicine, Mashhad University of Medical Sciences, Mashhad, Iran; 4 Pharmaceutical Research Center, School of Pharmacy, Mashhad University of Medical Sciences, Mashhad, Iran

**Keywords:** Choroid, Coronavirus disease 2019, Macula, Retina, Severe acute respiratory syndrome Corona virus 2, Spectral-domain optical coherence tomography

## Abstract

**Objectives:**

This study aimed to evaluate the retinal and choroidal changes in the macular region of patients with Coronavirus Disease 2019 (COVID-19) using structural spectral-domain optical coherence tomography (SD-OCT) analysis.

**Methods:**

This cross-sectional observational case-control study included patients recovered from COVID-19. The COVID-19 in all participants was confirmed using the reverse transcription-polymerase chain reaction (RT-PCR) technique. The participants had mild to moderate degree of disease without a history of hospitalization, steroid usage, or blood saturation below 92%. Macular SD-OCT was performed at least two weeks and up to one month after recovery from systemic COVID-19. Quantitative and qualitative changes detected by macular SD-OCT imaging were evaluated in COVID-19 recovered patients and compared with the results of age-matched normal controls.

**Results:**

Participants in this study included 30 cases (60 eyes) and 60 healthy controls (120 eyes). In total, 17 (28.3%) eyes in patient group showed at least one abnormal finding indicated by macular SD-OCT imaging included hyperreflective lesions in different retinal layers. In addition, dilated choroidal vessels and retinal pigment epitheliopathy were evident in 41 (68.3.6%) and 4 (6.6%) eyes in patient group, respectively, and their OCT findings resembled those with pachychoroid spectrum. No statistically significant differences were observed in retinal layers or retinal volume between the two groups. The mean ± SD subfoveal choroidal thickness (SFCT) was determined at 380.3 ± 12.40 μm, which was significantly thicker than that in control group (310.7 ± 57.5 μm) (*P* < 0.001).

**Conclusion:**

Regarding retinal thickness, no significant change was observed in different retina layers of patients with COVID-19; however, there were striking qualitative changes, such as hyperreflective lesions in different retinal layers. The evaluation of choroidal structure and thickness demonstrated remarkable abnormal pachyvessels and significant thickening of the SFCT but the clinical significance of these findings is unknown.

## Introduction

Severe Acute Respiratory Syndrome Coronavirus 2 (SARS-CoV-2) is a highly contagious infection with multi-organ involvement. Neurological manifestations have been reported throughout the disease course. Therefore, the potential involvement of the Central Nervous System (CNS) has been proposed in Coronavirus Disease 2019 (COVID-19) [1]. Since SARS-CoV-2 enters human cells by binding to the Angiotensin-Converting Enzyme (ACE2) 2 receptor, cells containing ACE2 are vulnerable to SARS-CoV-2 infection [2]. In addition to the lung alveolar cells, neurons, and glial cells in the CNS, several retinal components, including Müller cells, ganglion cells, retinal vascular endothelial cells (BRECs), and photoreceptor cells also contain ACE2 receptors [3]. Therefore, ocular tissue involvement is expected in the course of the disease. The most common and easily identifiable ocular manifestations of the SARS-CoV-2 have been reported in the ocular surface (e.g. conjunctival hyperemia, chemosis, and epiphora) [4–10].

Spectral-Domain Optical Coherence Tomography (SD-OCT) is a non-invasive imaging modality with high resolution that can be used for the cross-sectional evaluation of the retina. Several studies have been conducted on changes in choroidal and retinal structure, as well as thickness and vascularity worldwide; however, the results are very inconsistent [11–27]. This study aimed to evaluate the retinal and choroidal changes in the macular region of patients with COVID-19, both qualitatively and quantitatively, using structural SD-OCT analysis and compare the results with those obtained from a control group.

## Methods

### Study participants

This cross-sectional study was conducted in Imam Reza General Clinic, one of the largest centers devoted to COVID-19 patients in Mashhad, Iran. Patients recovered from COVID-19 were included in the study. All included subjects had a definite diagnosis of COVID-19 that had been confirmed through a positive real-time, reverse transcription-polymerase chain reaction (RT-PCR) assay as well as the test result of nasopharyngeal swab sample. The recovery period of these patients lasted from two weeks to not more than one month. Patients with a past medical history of diabetes mellitus, systemic hypertension, renal failure, proteinuria, or dementia, those with a history of any intraocular surgery and any significant refractive errors including pathologic myopia or high hyperopia, were excluded from the study. In addition, the patients with a history of more than mild diseases, including oxygen saturation less than 94% in room air at the course of the disease and those with any need for hospitalization or steroid usage were excluded to eliminate the confounding factors that might affect the study results. A control group was retrospectively selected from participants in an ongoing PERSIAN cohort study in Mashhad University of Medical Sciences, Mashhad, Iran [28]. The individuals in the control group were examined before January 2020, one month before the first report of a confirmed case of COVID-19 in the region.

### OCT image acquisition

The macular area of eligible subjects was imaged using SD-OCT (AngioVue, Optovue RTVue XR Avanti, Optovue, Fremont, CA). The device uses a laser beam with a wavelength of 840 nm and a scan rate of 70 kHz to obtain the images. Raster, Cross Line, Enhanced HD Line, and Retina Map protocols were used to evaluate the retina and choroid in the macular region of the patients. A panel of three experienced examiners (MA, MRAA, RD) evaluated each OCT image to pinpoint any retinal or choroidal finding and measure the subfoveal choroidal thickness (SFCT) and SD-OCT image segmentation accuracy. Any disagreement was resolved by discussion and consensus of all examiners. The SFCT was measured using the Crossline Images. Briefly, each horizontal or vertical foveal scan of the device is an average of at least twenty B-scan of the macular area with a width of 10 mm, and these horizontal and vertical ‘crosslines’ are centered at the fovea and perpendicular to each other. The ‘Caliper tool’ within Optovue machine software (version 2018.0.0.14) was used to measure the SFCT and determine the minimum distance between the outer aspect of Bruch’s membrane (identified as a hyperreflective line) and the sclera border (i.e., the innermost hyperreflective line of the chorioscleral interface). The measurement was repeated in both horizontal and vertical scans and the average was considered to determine SFCT. Pachyvessles that is seen in the spectrum of pachychoroid is defined as dilatation of the large choroidal vessels compressing the overlying choriocapillaris and Sattler’s layer. Pachychoroid pigment epitheliopathy is defined by OCT as numerous, scattered small elevations of the RPE representing RPE hyperplasia and sub-RPE drusen-like deposits and/or small serous PEDs [29]. Raster and Retina Map protocols of the device software were used to evaluate the following retinal thicknesses in the macular area: full retina (internal limiting membrane [ILM)] to Bruch membrane [BRM]), inner retina (ILM to the interface of the inner plexiform layer [IPL] and the inner nuclear layer [INL]), and outer retina (interface of the IPL and INL to BRM). The macular area was separated into three concentric circles: the fovea (central circle, with a diameter of 1 mm), the parafovea (inner diameter of 1 mm and outer diameter of 3 mm) and the perifovea (inner diameter of 3 mm and outer diameter of 5 mm). All subjects in both groups were examined using the same machine. All images with a signal strength index (SSI) of less than 50 were excluded. For each subject, the SD-OCT image of the eye with better SSI was included. The readers were blind on the status of the patients.

### Statistical analysis

Statistical analysis was performed using the SPSS software (Version 20; IBM SPSS Statistics, IBM Corporation, Chicago, IL, USA). Quantitative and categorical variables were presented with descriptive statistics (by providing central tendency measures for continuous variables) and frequencies. The normal distribution of variables was evaluated using the Shapiro-Wilk test and normality plots. Based on the data distribution, the independent samples t-test or Mann-Whitney U test was used to compare thickness measurements between the independent samples (control and case groups). A *p*-value less than or equal to 0.05 was considered statistically significant for all tests.

### Ethical considerations

The study protocol adhered to the tenets of the 1964 Declaration of Helsinki and its later amendment. The written informed consent was obtained from all participants in the study, and participation in this study was voluntary and based on willingness. The study protocol was approved by the Medical Ethics Committee at the Mashhad University of Medical Sciences, Mashhad, Iran (IR.MUMS.REC.1399.104).

## Results

This study was conducted on 60 eyes of 30 patients (52.9% male) with the mean ± SD age of 39.6 ± 1.7 years, and 120 eyes of 60 healthy controls (31 males) with a mean ± SD age of 39.3 ± 7.7 years. There was no significant difference between the two groups in terms of age (*P* = 0.263) and gender (*P* = 0.635). The mean ± SD SSI was 75.7 ± 6.8 in the COVID-19 cases and 72.3 ± 8.3 in the normal controls (*P* = 0. 181).

### Macular retinal analysis

**Fig. 1:**
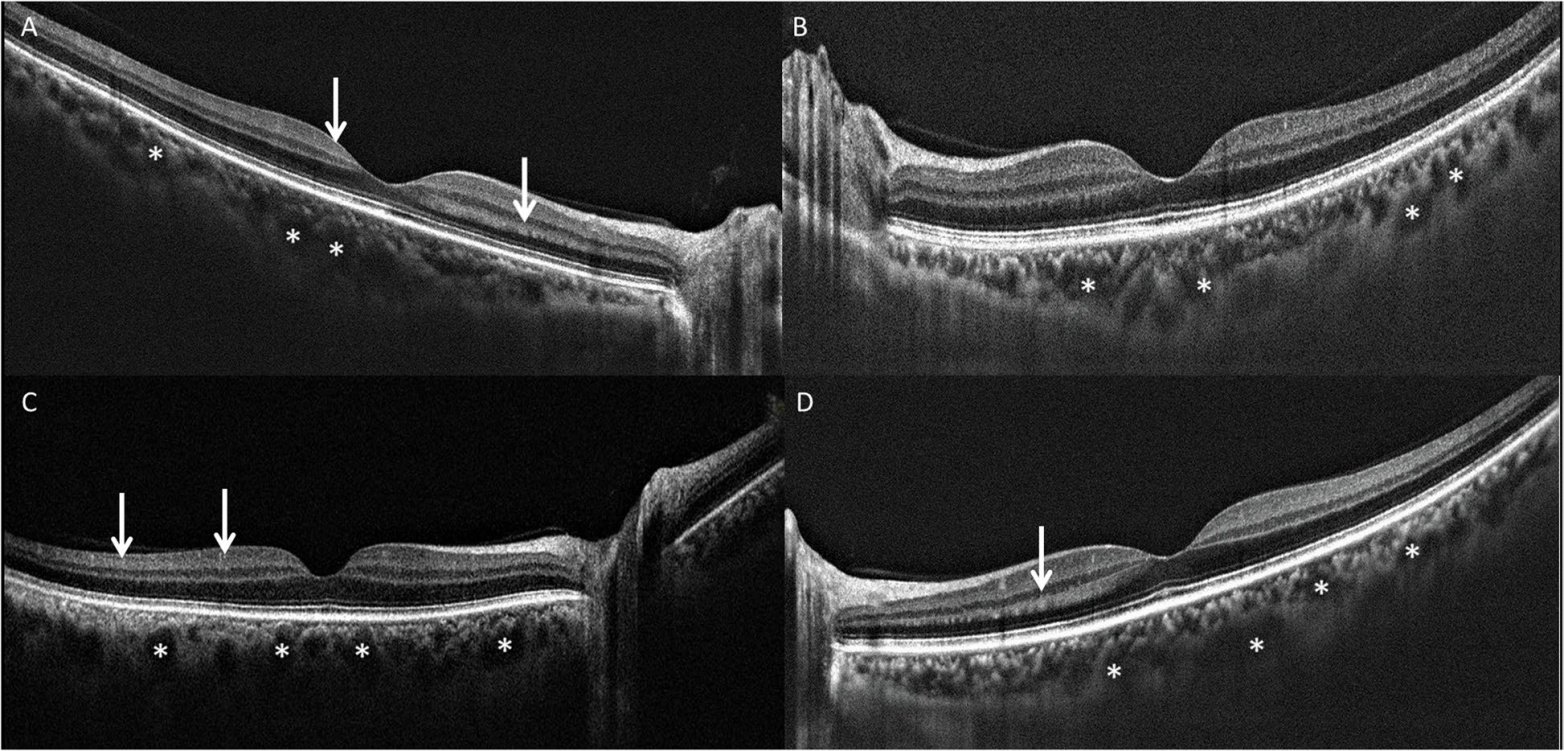
Spectral domain optical coherence tomography finding of macular area in a representative patient with coronavirus disease 2019. Note the hyperreflective bands in different retinal layers (white arrows) and dilated choroidal vessels (asterisks)

**Fig. 2:**
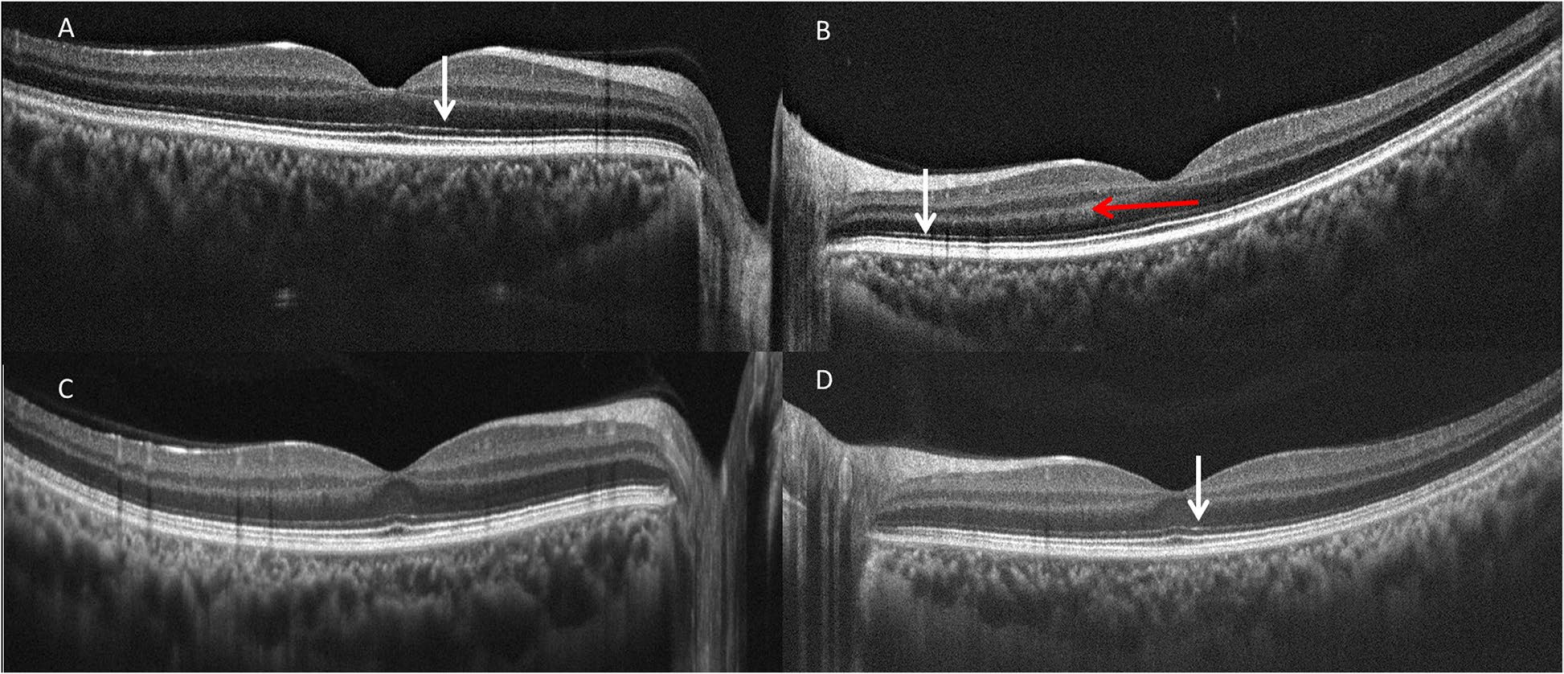
Spectral domain optical coherence tomography finding of macular area in a representative patient with coronavirus disease 2019. Note marked thickening of the choroid, the hyperreflective bands in outer nuclear layer/ outer plexiform layer (red arrow) and the external limiting membrane (vertical white arrow)

**Fig. 3:**
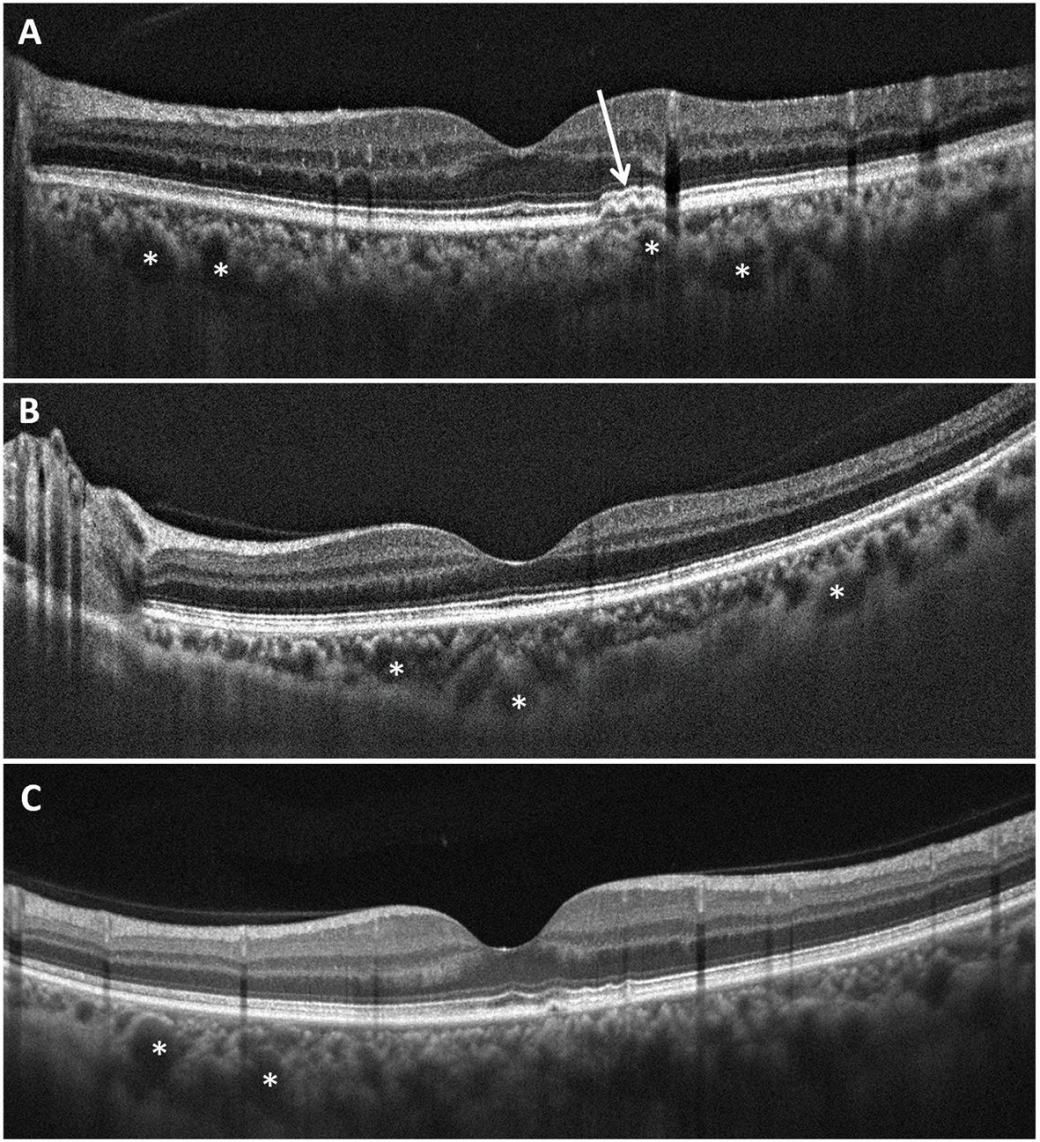
Spectral domain optical coherence tomography finding of macular area in a representative patient with coronavirus disease 2019. Note the dilated choroidal vessels (asterisks), and overlying retinal pigment epitheliopathy (white arrows). The findings are similar to those in pachychoroid spectrum

At least one abnormal finding, such as hyper reflective lesions, was observed in the images of 17 eyes (28.3%) in different retinal layers in patient group. Qualitatively, these SD-OCT features included hyperreflective lesions in retinal ganglion cell layer (GCL) (*n* = 11, 18.3%), retinal nerve fiber layer (RNFL) (*n* = 7, 11.7%), outer plexiform layer (OPL) (*n* = 5, 8.3%), inner plexiform layer (IPL) (*n* = 4, 6.7%), inner nuclear layer (INL) (*n* = 4, 6.7%), outer nuclear layer (ONL) (*n* = 2, 3.3%), external limiting membrane (ELM) (*n* = 1, 1.7%) (Figs. 1, 2, and 3).

As shown in Table 1, the difference in the full retinal thickness of the case and control groups in parafoveal (311.2 ± 19.0 vs. 308.9 ± 14.1, respectively; *P* = 0.606) and perifoveal (285.4 ± 16.3 vs. 282.7 ± 10.1, respectively; *P* = 0.142) rings were not statistically significant. A not statistically significant increase was also observed in the inner (parafovea: 129.1 ± 9.6 vs. 128.1 ± 7.4, *P* = 0.629; perifovea: 113.9 ± 8.1 vs. 112.7 ± 5.3, *P* = 0.200) and outer (parafovea: 181.9 ± 10.5 vs. 180.7 ± 8.7, *P* = 0.559; perifovea: 171.6 ± 8.9 vs. 170.1 ± 6.7, *P* = 0.175) retinal thicknesses that were not statistically significant (Tables 2, 3). It is worth mentioning that no statistically significant difference in foveal thickness was observed between the case and control groups in inner, outer, or full retinal thickness analysis. Moreover, retinal volumes were also increased in different layers of the retina that was not statistically significant (Tables 1, 2, and 3).

**Table 1:** Comparison of the Full Retinal Thickness between the COVID-19 Patients (*N* = 25) and Control Group (*N* = 60)

Variable	Retinal Thickness			Retinal Volume		
	Control	Case	***P***-value	Control	Case	***P***-value
**Fovea**	245.7 ± 20.0	248.3 ± 20.5	0.407	0.193 ± 0.016	0.195 ± 0.016	0.415
**Parafovea**	308.9 ± 14.1	311.2 ± 19.0	0.606	1.940 ± 0.088	1.954 ± 0.119	0.633
S Hemisphere	308.7 ± 13.7	312.2 ± 18.6	0.417	0.970 ± 0.043	0.981 ± 0.058	0.423
I Hemisphere	308.9 ± 14.9	310.1 ± 19.5	0.794	0.971 ± 0.047	0.974 ± 0.061	0.813
Temporal	299.5 ± 14.6	303.8 ± 19.2	0.265	0.470 ± 0.023	0.485 ± 0.045	0.137
Superior	312.6 ± 13.0	315.3 ± 18.4	0.46	0.491 ± 0.020	0.495 ± 0.029	0.429
Nasal	311.7 ± 16.5	314.1 ± 19.2	0.654	0.490 ± 0.026	0.493 ± 0.030	0.63
Inferior	311.6 ± 14.7	311.2 ± 20.5	0.965	0.489 ± 0.023	0.489 ± 0.032	0.923
**Perifovea**	282.7 ± 10.1	285.4 ± 16.3	0.142	3.552 ± 0.127	3.587 ± 0.204	0.147
S Hemisphere	284.6 ± 10.1	287.9 ± 16.2	0.124	1.788 ± 0.064	1.809 ± 0.101	0.117
I Hemisphere	280.8 ± 10.8	283.0 ± 16.9	0.257	1.764 ± 0.068	1.778 ± 0.106	0.267
Temporal	271.9 ± 11.1	276.0 ± 15.4	0.077	0.853 ± 0.035	0.867 ± 0.048	0.056
Superior	284.3 ± 10.4	287.8 ± 17.1	0.089	0.893 ± 0.033	0.904 ± 0.053	0.093
Nasal	298.1 ± 11.7	299.9 ± 17.7	0.518	0.936 ± 0.037	0.942 ± 0.055	0.475
Inferior	276.4 ± 11.2	278.1 ± 17.3	0.377	0.868 ± 0.035	0.874 ± 0.054	0.357

Notes: The unit of measurement thicknesses and volumes were μm and mm^3^, respectively. Values represented as mean ± SD and analysis made by Mann-Whitney U test

Abbreviations: *S* superior, *I* inferior

**Table 2:** Comparison of the Inner Retinal Thickness between the COVID-19 Patients (*N* = 25) and Control Group (*N* = 60)

Variable	Retinal Thickness			Retinal Volume		
	Control	Case	***P***-value	Control	Case	***P***-value
**Fovea**	67.8 ± 11.4	68.6 ± 12.3	0.675	0.053 ± 0.009	0.054 ± 0.010	0.616
**Parafovea**	128.1 ± 7.4	129.1 ± 9.6	0.629	0.805 ± 0.046	0.811 ± 0.060	0.643
S Hemisphere	127.7 ± 7.7	129.2 ± 9.4	0.38	0.401 ± 0.024	0.406 ± 0.030	0.367
I Hemisphere	128.5 ± 7.9	128.9 ± 10.2	0.828	0.403 ± 0.026	0.405 ± 0.032	0.721
Temporal	119.5 ± 8.3	120.2 ± 9.3	0.595	0.188 ± 0.013	0.189 ± 0.015	0.609
Superior	131.0 ± 8.1	131.9 ± 9.8	0.626	0.206 ± 0.013	0.207 ± 0.015	0.599
Nasal	130.4 ± 8.8	133.5 ± 11.4	0.191	0.205 ± 0.014	0.210 ± 0.018	0.193
Inferior	131.4 ± 8.4	130.6 ± 11.0	0.946	0.206 ± 0.013	0.205 ± 0.017	0.981
**Perifovea**	112.7 ± 5.3	113.9 ± 8.1	0.2	1.415 ± 0.067	1.430 ± 0.103	0.193
S Hemisphere	112.8 ± 5.6	114.3 ± 8.4	0.195	0.709 ± 0.035	0.718 ± 0.053	0.182
I Hemisphere	112.4 ± 5.5	113.3 ± 8.3	0.279	0.707 ± 0.035	0.712 ± 0.052	0.309
Temporal	106.3 ± 6.3	107.4 ± 7.5	0.268	0.334 ± 0.020	0.337 ± 0.024	0.313
Superior	112.0 ± 5.8	112.8 ± 8.6	0.379	0.352 ± 0.018	0.354 ± 0.027	0.404
Nasal	121.7 ± 8.1	124.8 ± 9.9	0.133	0.383 ± 0.026	0.392 ± 0.031	0.127
Inferior	110.0 ± 5.4	110.4 ± 8.6	0.49	0.346 ± 0.017	0.346 ± 0.027	0.521

Notes: The unit of measurement thicknesses and volumes were μm and mm^3^, respectively. Values represented as mean ± SD and analysis made by Mann-Whitney U test

Abbreviations: *S* superior, *I* inferior

**Table 3:** Comparison of the Outer Retinal Thickness between the COVID-19 Patients (*N* = 25) and Control Group (*N* = 60)

Variable	Retinal Thickness			Retinal Volume		
	Control	Case	***P***-value	Control	Case	***P***-value
**Fovea**	177.9 ± 11.3	179.3 ± 11.3	0.71	0.140 ± 0.009	0.141 ± 0.009	0.717
**Parafovea**	180.7 ± 8.7	181.9 ± 10.5	0.559	1.136 ± 0.054	1.143 ± 0.066	0.589
S Hemisphere	181.0 ± 9.2	182.8 ± 10.9	0.566	0.569 ± 0.029	0.574 ± 0.034	0.524
I Hemisphere	180.5 ± 8.9	181.0 ± 10.6	0.728	0.567 ± 0.028	0.568 ± 0.033	0.78
Temporal	179.9 ± 10.0	183.6 ± 13.1	0.313	0.283 ± 0.016	0.288 ± 0.021	0.339
Superior	181.5 ± 9.2	183.0 ± 11.0	0.54	0.285 ± 0.014	0.288 ± 0.017	0.537
Nasal	181.2 ± 10.9	180.6 ± 10.2	0.761	0.285 ± 0.017	0.284 ± 0.016	0.75
Inferior	180.3 ± 8.9	180.4 ± 11.2	0.981	0.283 ± 0.014	0.283 ± 0.017	0.996
**Perifovea**	170.1 ± 6.7	171.6 ± 8.9	0.175	2.137 ± 0.085	2.156 ± 0.111	0.168
S Hemisphere	171.8 ± 7.3	173.6 ± 8.8	0.143	1.079 ± 0.046	1.091 ± 0.056	0.14
I Hemisphere	168.4 ± 7.0	169.4 ± 9.3	0.377	1.057 ± 0.044	1.065 ± 0.058	0.328
Temporal	165.5 ± 6.9	168.8 ± 9.4	0.078	0.518 ± 0.023	0.530 ± 0.030	0.054
Superior	172.3 ± 7.7	174.9 ± 9.7	0.08	0.541 ± 0.024	0.550 ± 0.031	0.073
Nasal	176.1 ± 10.5	174.9 ± 9.4	0.881	0.553 ± 0.033	0.549 ± 0.030	0.9
Inferior	166.3 ± 7.2	167.6 ± 9.9	0.372	0.522 ± 0.023	0.527 ± 0.031	0.367

Notes: The unit of measurement thicknesses and volumes were μm and mm^3^, respectively. Values represented as mean ± SD and analysis made by Mann-Whitney U test

Abbreviations: *S* superior, *I* inferior

### Macular choroidal analysis

**Fig. 4:**
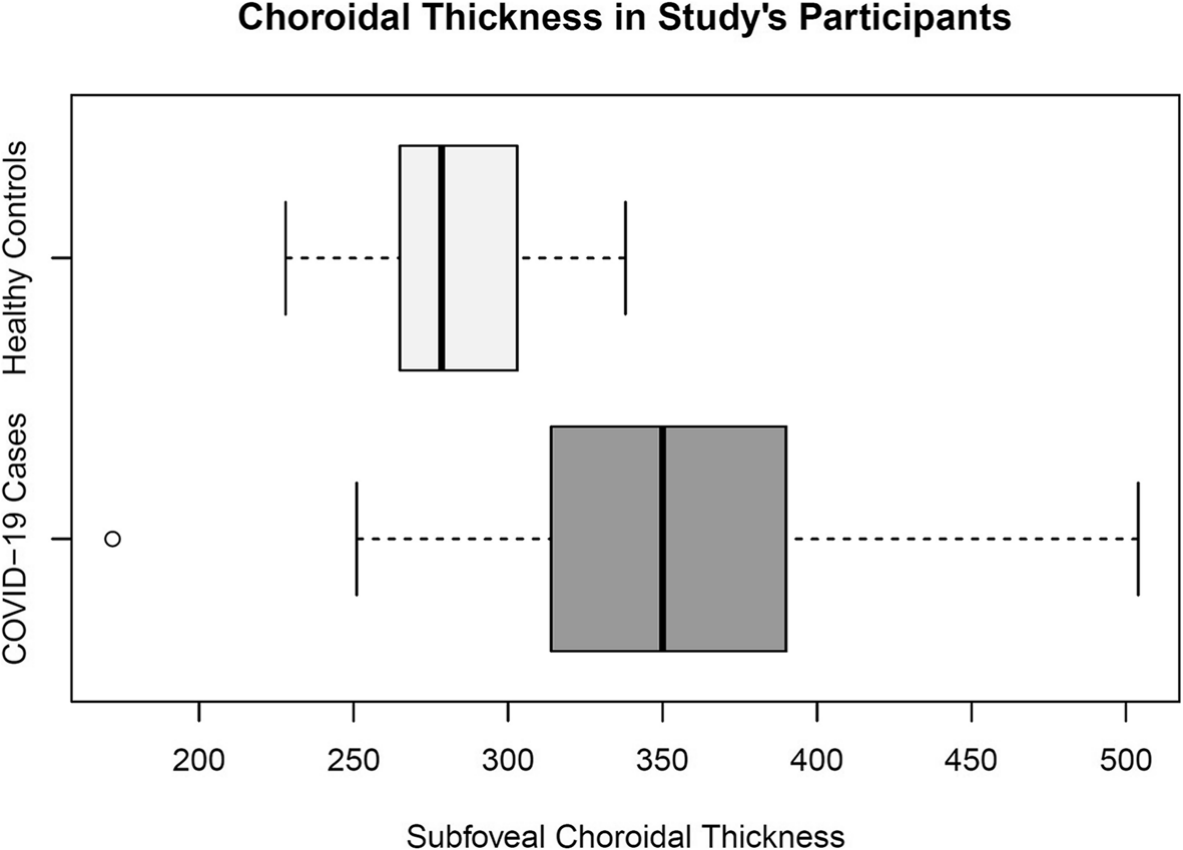
Box plots depict choroidal thickness in COVID-19 patients and healthy controls

Some symptoms of pachychoroid spectrum disorder, including dilated choroidal vessels (i.e. pachyvessels and retinal pigment epitheliopathy), were observed in patient group. Dilated choroidal vessels were observed in 48 (71.6%) eyes and retinal pigment epitheliopathy was evident in 4 (6.0%) eyes (Fig. 1). The mean SFCT (380.3 ± 12.40 μm) was significantly thicker, compared to the control group [28] (310.7 ± 57.5 μm) (*P* < 0.001) (Fig. 4).

## Discussion

In this study, the macular SD-OCT findings in retinal and choroidal layers of patients recovered from COVID-19 were evaluated, and subfoveal choroidal, as well as inner-, outer-, and full- retinal thickness and volume of the recovered COVID-19 patients were compared with those in healthy control subjects. Hyperreflective lesions in all retinal layers and unremarkable incremental patterns in macular retinal thickness and volume were noted in patients with a history of COVID-19. Some features of pachychoroid spectrum disorder, including pachyvessels, retinal pigment epitheliopathy, and significantly thicker SFCT was also observed in these patients.

Marinho et al. were the first investigators to report abnormal retinal OCT findings in patients (*n* = 12) with COVID-19 [11]. They noticed hyperreflective lesions at the level of GCL and IPL, and also reported fine Cotton-Wool Spots (CWS) and microhemorrhages along with the retinal arcades in four patients, suggesting an inflammatory or ischemic process. In a later editorial article, Vavvas et al. raised some concerns regarding the interpretation of the OCT and fundus findings in COVID-19 patients [12]. They suggested that as CWS were very subtle and could be due to some comorbid conditions, the results provided by Marinho et al. should be interpreted with caution. Since then numerous and interesting studies have been published on the subject of OCT and OCTA findings in the COVID-19 patients [13–27]; however, the results obtained in these studies have been very different and sometimes contradictory. The more time passes from the onset of this epidemic, the more we know about the specific effects of this disease on the neurosensory tissues of the eye. In this study, the detailed SD-OCT quantitative and qualitative analysis of the retinal and choroidal structure and thickness in patients with a recent history of COVID-19 was reported and the obtained data were compared with those from the healthy control group. One of the strengths of the present study is the elimination of all severe cases of the disease. Hospitalization, steroid consumption, the need for oxygen, and multiorgan failure were all confounding and destructive factors that might have affected the results.

SARS-CoV-2 replication will be initiated after binding to epithelial cells in the nasopharynx and the nasal cavity. ACE2 has been found as the main receptor for SARS-CoV-2 [30]. ACE2 is an enzyme in cell membranes of type II alveolar cells of the lung, enterocytes of the small intestine, arterial and venous endothelial cells, and arterial smooth muscle cells in most organs [31]. The ACE2 has been found in humans, rodents, and the porcine retina. Moreover, the ACE has been reported to be present in the choroid and different cell types of the retina, including Müller cells, ganglion cells, BRECs, and photoreceptor cells. Therefore, the retina and choroid are well anticipated to be potential targets of SARS-CoV-2 infection [5].

Some hyperreflective lesions were observed in all retinal layers as reported by Marinho et al. [11]. However, as suggested by Vavvas et al. [12], it is believed that these hyperreflective bands are probably the abnormal and prominent retinal vessels, and the prominence of these findings in COVID-19 patients could be due to vascular involvement (i.e., microthrombosis and vasodilation) associated with this infection. This has been confirmed in recent autopsy specimens, demonstrating vascular dilation and intravascular microthrombosis in these patients [32]. Although hyper reflective foci or dots can be retinal vesseles section in OCT but these hyper reflective bands have been seen in different retinal layers and all of them can’t be retinal vessels. They have been reported in different retinal diseases, and could be inflammatory activated and swelled cells,activated microglia cells,focal accumulations of pigment or lipofuscin granules or small intraretinal proteins or lipid exudates/deposits due to the breakdown of the blood-retinal barrier [33].

Reinhold et al. also reported fibrin microthrombi in choroidal microvasculature, endothelial swelling, and congestion of choroidal vessels in COVID-19 patients. Moreover, some features of the pachychoroid spectrum of disorders were noted in this study, including increased SFCT, presence of pachyvessels, and retinal pigment epitheliopathy. Based on the results of previous studies, murine coronavirus induced a biphasic retinal disease in adult mice. The early phase of this disease was associated with inflammation, including retinal vasculitis and viral replication, and the late phase was associated with retinal degeneration [34–36]. Therefore, it can be hypothesized that choroidal thickening in COVID-19 patients may happen in two phases, including an initial inflammatory phase with increased vascular permeability followed by a late apoptotic phase with infiltration of the retina by inflammatory cells. However, there is no direct evidence for this claim. Increased choroidal thickness in COVID-19 cases can be attributed to choroidal vascular dilation as an autoregulatory response to hypoxia and hypercapnia. The mean SFCT in our patients was almost 70 μm higher than that in normal subjects. Despite the limited accuracy of SFCT measurement, Rahman et al. reported that a change in SFCT more than 32 μm is more than interobserver variability and could be a real change [37].

The results of previous studies conducted on structural and vascular changes in choroid and macula are very different and inconsistent. Burgos-Blasco et al. [13] investigated the peripapillary and macular layer thicknesses in 90 recovered COVID-19 patients and found decreases in macular RNFL and an increase in GCL thickness in COVID-19 patients. Cetinkaya et al. [14] selected the hospitalized patients with COVID-19 and treated them with favipiravir, moxifloxacin, and heparin without the need for intubation. The SS-OCT measurements showed no retinal neurodegenerative and choroidal thickness alterations; therefore, they concluded that insignificant results might be due to the examination of the patients in the early period of the COVID-19 after treatment. Their results differed from those obtained in the present study in terms of choroidal thickness and not retinal thickness. Oren B, et al. [15] evaluated 35 patients in the COVID-19 group 14–30 days after the onset of the COVID-19 symptom. In their study, the mean value of central macular thickness was significantly high despite the thinner ganglion cell layer and inner nuclear layer. They did not found any significant change in RNFL thickness. González-Zamora et al. [16] found thinner ganglion cell layer (GCL) and thicker RNFL, compared to controls in their study. Results of a study conducted by Hepokur M., et al. [24] was inconsistent with those obtained in the present study and other studies in terms of choroidal thickness, and they found that choroidal thickness was reduced in all measured areas and this decrease affected all choroidal layers. Fırat M [25] evaluated the treatment of COVID-19 patients without comorbidities and concluded that choroidal and retinal thicknesses were not affected in patients with recent mild COVID-19. Their study was similar to the present study in the term of patients’ selection; however, it was different in the term of choroidal thickness changes in the patients. Dominika et al. [26] evaluated 156 eyes of post-COVID-19 patients and 98 eyes of subjects. The OCT examination did not detect any significant changes in morphology or morphometry of the optic nerve, retina, or retina vessels due to COVID-19. These multiple studies with different results indicate the importance of a large sample size study that considers multiple confounding factors (e.g., disease severity) into account, in certain stages of the disease.

Despite different results obtained in different studies conducted on retinal and choroidal layers and thickness based on OCT assessment, there is more consensus on reduced vascular density in the macular region, according to OCTA findings. Decreased vascular density in macula was found in multiple studies, and they have concluded that reduced vessel density of retinal capillary plexus detected in COVID-19 may result in retinal vascular complications and microvascular alterations in COVID-19 patients [16–23].

Regarding the limitations of the present study, it should be noted that due to conditions related to COVID-19 pandemic limitations and restrictions we didn’t able to evaluate retinal and choroidal findings changes overtime. In two other cohort studies, longitudinal changes were evaluated by authors [38, 39]. Based on the results, the patients who had recovered from COVID-19 had a progressive decrease of vessel density at the follow-up visit 3 months after COVID-19 infection and choroidal vascular index is increased in patients with COVID-19, one month after recovery from COVID-19 and returns to baseline values after three months. Another limitation was not obtaining OCT images during the acute phase of the disease. Moreover, this study had a relatively small sample size which might justify the insignificant findings and is likely not powered enough to detect statistical significance. Furthermore, since the control individuals were selected retrospectively from a large sample of an eligible cohort, selection bias might have occurred during the identification of the control arm. The OCT machine in this study had a limited resolution, especially for the evaluation of choroidal changes. The application of swept-source OCT devices can potentially yield more helpful information. A larger-scale study during the acute phase of the disease, followed by repeated exams at fixed intervals would provide valuable information about the impact of COVID-19 on the retina and choroid, considering the diversity of COVID-19. Finally, it is necessary to mention that the clinical significance of these findings is unknown.

In conclusion, the results of this study showed that patients with COVID-19 developed not statistically significant thickening in retinal layers which can demonstrate choroidal changes similar to that in patients with the pachychoroid spectrum. It was also found that in COVID-19 patients, hyperreflective lesions are present in all retinal layers and the findings of the current study indicate that retina and choroid could be involved in patients with COVID-19. These findings highlight the necessity of comprehensive ophthalmic examinations in these patients.

## Data Availability

The datasets used and analyzed during the current study are available from the corresponding author on reasonable request.
